# Unlocking efficiency in column chromatography with packed bed supporting inserts

**DOI:** 10.3389/fbioe.2025.1613174

**Published:** 2025-06-05

**Authors:** Romone M. Fancy, David H. Abraham, Matthew R. Taylor, Cole McMullin, Nicholas S. Brann, Daniel M. Bailey, David I. Brown, Heather Bethea Horne, Leslie S. Wolfe

**Affiliations:** ^1^ KBI Biopharma, Durham, NC, United States; ^2^ JSR Life Sciences, Sunnyvale, CA, United States

**Keywords:** resin bed, chromatography, biomanufacturing, antibody, purification, permeability, column insert, hydraulic radius

## Abstract

To purify increasing amounts of biotherapeutics more efficiently, the use of high flow rates or greater resin bed heights during downstream chromatography steps is a tantalizing option. A limitation of utilizing high flow rates is the differential pressure generated by packed chromatography resin beds. As a resin bed height increases, the resin is susceptible to compression. By increasing the permeability of a packed resin bed through control of the hydraulic radius, column pressure-flow dynamics can be improved. Chromatography column performance using a commercially available Protein A resin was assessed with and without OMEGA, a column insert designed to modulate the hydraulic radius of the column by providing vertical supports through the packed resin bed. OMEGA was shown to reduce the effective hydraulic radius of packed resin beds, increase the permeability of packed columns by 44%–73%, and yield a 42%–50% decrease in pressure differential across the resin bed at a comparable linear velocity. The structural support provided by OMEGA enables higher operational flow rates and increased resin bed height without impact to either dynamic binding capacity or purified product quality. With the OMEGA column insert, scale-up hurdles are mitigated, and faster downstream processing times are unlocked across column geometries.

## Introduction

As cell culture titers of biotherapeutics are driven to higher levels, a greater burden is placed on downstream processing to achieve a faster and more cost-effective operation. Leveraging higher flow rates and larger scale chromatography columns are approaches that may be used to reduce downstream processing time and control costs by minimizing manufacturing suite time; however, chromatography resins impose pressure limitations, restricting flow rates to well under the maximum capacity of manufacturing suite liquid handlers. Due to the compressible nature of resin chromatography beads, increases in flow rate, resin bed height, or column diameters result in dramatic increases in pressure differential (ΔCP) within the column (Stickel and Fotopoulos, 2001). This column ΔCP must be avoided to prevent damage to chromatography resin beds and associated processing equipment. Despite the capacity for increased flux with liquid handlers, elevated flow rates have remained inaccessible.

The development of resin-based chromatography steps for operation at the manufacturing-scale typically begins with columns of inner diameters (ID) of 1–5 cm. At the commercial manufacturing-scale, a larger volume of resin is needed, which requires the use of larger columns. The increase in column size is typically accomplished by increasing column diameter rather than bed height; commercial offerings for column hardware can reach up to 200 cm in inner diameter, while the resin bed height is typically limited to 30 cm to avoid compression (Łącki, 2018). Despite maintaining bed height when transitioning from development-scale to manufacturing-scale columns, column ΔCP hurdles are commonly encountered necessitating a reduction in flow rate. Increasing inner diameter results in a dramatic change in column aspect ratio and an increase in the hydraulic radius. In the case of an open channel, an increase in hydraulic radius increases the volumetric flow rate of a fluid channel due to a reduction in drag; however, when applied to a packed resin bed, an increase in hydraulic radius often requires a reduction in volumetric flow rate to avoid excessive column ΔCP. This is partially attributable to wall effects: resin near the center of a cylindrical column experiencing compression due to the lack of frictional support provided by the column walls (Colby, O’Neill, and Middelberg, 1996; Soriano, Titchener-Hooker, and Shamlou, 1997; Parker, Mehta, and Caro, 1987; Guiochon et al., 1999). The compression at the center of a packed resin bed results in decreased permeability and a flow velocity gradient across the diameter of the column. As the column diameter and hydraulic radius increases, a greater proportion of the resin bed is susceptible to compression resulting in decreased permeability (Prentice et al., 2020). With decreased permeability, a column’s resolving power and theoretical plate count are reduced (“Consolidation of Particle Beds and Packing of Chromatographic Columns,” n.d.).

Performance enhancements of modern resin-based chromatography operations have included improvements to resin matrices and advanced ligand chemistries. These improvements have yielded superior stability and packing performance relative to traditional chromatography resins yet are still susceptible to performance degradation resulting from compression. One existing substitute for resin-based chromatography is monolithic columns, which eliminate column packing and can operate at higher flow rates. The major drawbacks of monolithic columns are the limited choice of column chemistries and dimensions as well as their lower binding capacities relative to chromatography resins (Rathore, Kumar, and Kateja, 2018). An emerging alternative to resin chromatography in downstream processing is membrane-based adsorption technology (Qu et al., 2023). Membrane adsorbers are capable of operation at much higher flow rates; however, due to the limited binding capacity of membrane adsorbers available on the market today, an increased number of processing cycles are required relative to resin chromatography to process the same amount of biotherapeutic (Winderl, Neumann, and Hubbuch, 2021; Liu et al., 2011). At this time, it is uncertain if wide-scale adoption of membrane absorption technologies are viable at commercial scale operations (Qu et al., 2023). Strategies to enhance the performance of existing resin media include rapid cycling of short bed-height columns in batch mode (Kaltenbrunner et al., 2016; Guo, Jin, and Kanani, 2020), thereby increasing the productivity (grams of product processed per mL of resin per hour) of resin batch operations. However, rapid cycling in short bed-height resin batch chromatography lowers the resin capacity utilization, which makes inefficient use of resin lifetime and process buffers. Methods designed to use multiple loading flow rates to increase resin utilization capacity while maintaining high process productivity have also been reported (Kanani, 2018; 2019) and modeled (Guo, Jin, and Kanani, 2020). Continuous multicolumn chromatography has also been explored to process more load material using less resin (Kaltenbrunner et al., 2016), has been demonstrated to be feasible at the pilot scale (Ötes et al., 2018), and similarly modeled for process productivity (Guo, Jin, and Kanani, 2020). While the use of multiple flow rates and multiple short bed-height columns has great potential, these technologies do not fully address concerns with resin compression, and hurdles remain for their implementation at manufacturing scale. A technology that provides a performance upgrade to existing standard resin batch mode operations, while also maintaining compatibility for use with future continuous modes of operation is needed.

Reducing the compressibility of chromatography resins can be achieved by providing additional wall support with column inserts. When reducing the amount of compression experienced throughout a resin bed by use of column inserts consisting of concentric rings, simulations of packed columns have shown that the permeability of a packed resin bed is increased (Gerontas et al., 2015; Riess et al., 2022). According to Darcy’s Law, increased permeability enables the use of higher flow rates or increased resin bed heights without a corresponding increase in ∆CP across the resin bed. A concentric ring column insert design does not directly control the column’s hydraulic radius and imposes practical difficulties for column packing. By managing a column’s hydraulic radius through tuned column inserts, we hypothesize that permeability will be controllable independent of column geometry. With regulated resin bed permeability, higher flow rates may be achievable while maintaining the resin’s performance characteristics. This would enable large-scale columns to have pressure-flow characteristics similar to small-scale columns while retaining the increased binding capacity associated with a scale-up in the volume of resin.

**FIGURE 1:**
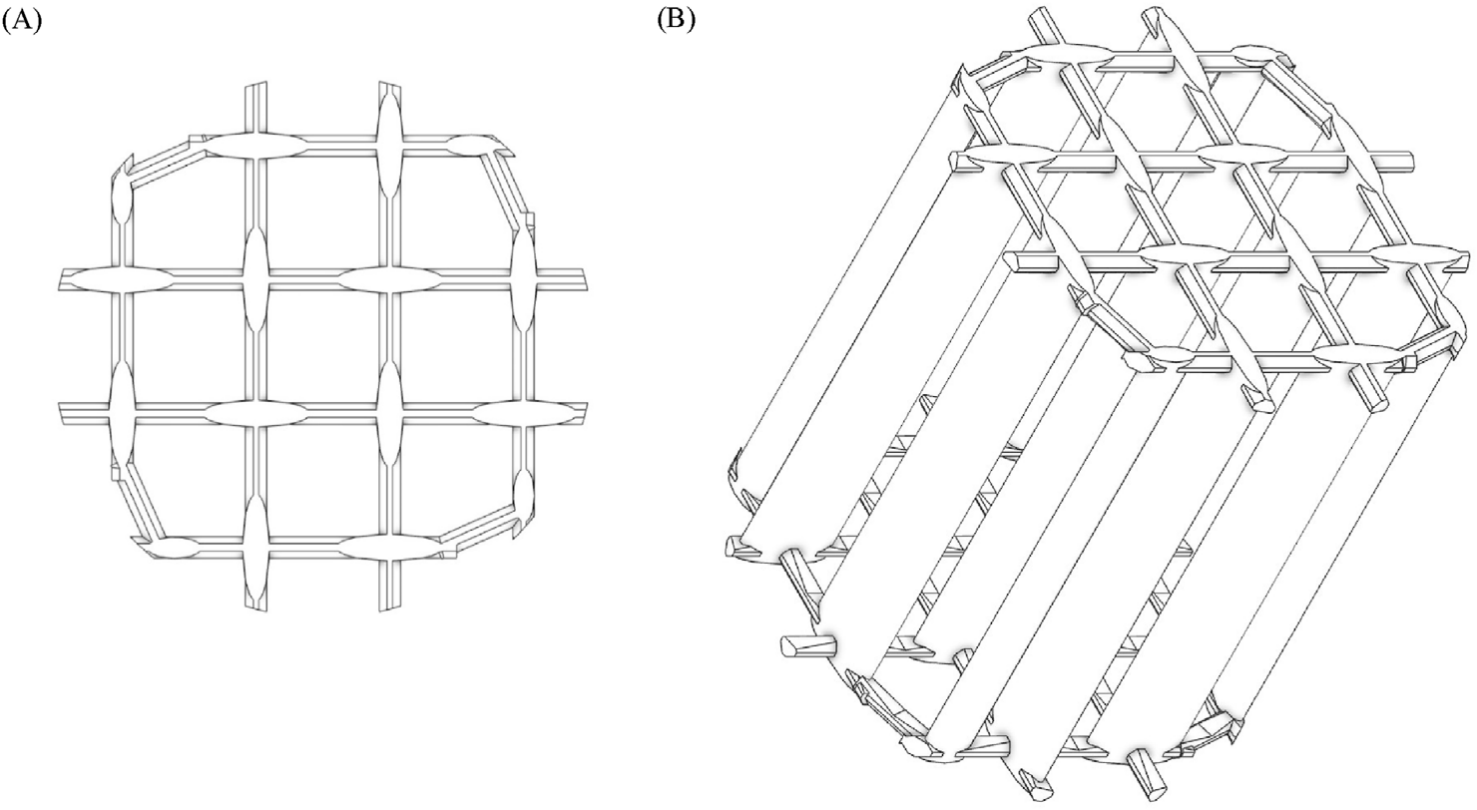
Schematics of an OMEGA device at a top-down view and isometric view. **(A)**: Top-down view. **(B)**: Isometric view.

In this work, we evaluate a novel design for a column insert (OMEGA) that reduces the hydraulic radius of a resin bed by providing vertical wall supports throughout the full height of the bed. This degree of support addresses the unmet need for improved processing time, maintains resin bed integrity, and allows for simple column packing (Figure 1). As designed, OMEGA reduces the hydraulic radius in a manner that is decoupled from the column’s inner diameter. Effectively, the amount of support provided for each column geometry is scaled according to the inner diameter of the column. The degree of compression alleviated by OMEGA’s inclusion in a packed resin bed was measured across several column geometries. The resultant change in compression and its impact on permeability was assessed in this work. With and without the use of OMEGA within columns, the impact of permeability on operational flow rates and ∆CP was measured. Lastly, a model monoclonal antibody (mAb) was purified using a traditional chromatography column and a column supported with OMEGA inserts. Purification performance of the model mAb was analyzed for column operational characteristics as well as product quality.

## Materials and methods

### Column insert design

The column insert (OMEGA) as used in this study is a polystyle structure containing vertical structural members, perpendicularly oriented to each other as shown in the schematic in Figure 1. The spacing, shape, and distribution of the vertical supports were designed to modulate the hydraulic radius of the packed resin bed to maintain a more consistent hydraulic radius across column scales (Bailey and Guido, 2024). The column insert for the 2.6 cm ID column had an inner diameter of 2.6 cm, a height of 5 cm, and a total volume of 3.8 mL. The 14 cm ID column insert had an inner diameter of 14 cm, a height of 5 cm and a total volume of 106.8 mL. Column inserts were stacked incrementally to accommodate every 5 cm of packed bed height.

### Column and resin selection

Assessment of column performance was performed with and without OMEGA for 2.6 cm ID and 14 cm ID columns; additionally, a 1.6 cm ID column was used to represent a development-scale column. A commercially available Protein A resin was used throughout column performance and purification assessments. Additional consideration was made to select a widely available resin with known pressure limitations that emerge during process scaling, which is easily deployable for a model mAb. This Protein A resin provides a consistent degree of impurity clearance which allows for an evaluation of the effects of the inclusion of OMEGA on a product quality profile.

### Determination of resin bed hydraulic radius

Hydraulic radius is defined as the cross-sectional area of an open channel divided by its wetted perimeter. As applied to a chromatography column, the wetted perimeter is the cross-sectional circumference of all points of contact between the resin bed and wall support which, in a traditional chromatography column, is only along the perimeter. For a chromatography column without inserts, hydraulic radius was calculated by Equation 1 (Wei, Cheng, and Lu, 2023):

$$R_{H}=\frac{\text{Cross}‐\text{sectional}\;\text{Area}\;\text{of}\;\text{Column}\;}{\text{Perimeter}\;\text{of}\;\text{Column}\;}$$ (1)

The hydraulic radius calculation was modified to account for the cross-sectional area displaced by the OMEGA insert from the total cross-sectional area of the column to yield the effective cross-sectional area of the resin bed. A detailed accounting of the contribution due to the OMEGA geometry was performed (Supplementary Material and Methods). Conversely, the cross-sectional perimeter of the OMEGA insert was added to the perimeter of the column wall to account for the increase in resin wall support provided by OMEGA. For columns which included OMEGA, hydraulic radius was calculated by Equation 2:

$$R_{H\;}=\frac{\left(\text{Cross}‐\text{sectional}\;\text{Area}\;\text{of}\;\text{Column}\;‐\;\text{Cross}‐\text{sectional}\;\text{Area}\;\text{of}\;\text{OMEGA}\right)\;}{\left(\text{Perimeter}\;\text{of}\;\text{Column}+\text{Perimeter}\;\text{of}\;\text{OMEGA}\right)\;}$$ (2)

### Column packing and column performance tests

All columns were packed with a commercially available Protein A resin under constant pressure flow conditions using an ÄKTA™ (Cytiva, Uppsala, Sweden) liquid handler system using 0.1 M NaCl. Columns were then equilibrated with 1M NaCl. Resolution and compression of packed resin beds were assessed with an injection spike of 2 M NaCl equal to 2% of the column volume. The conductivity peak area of the 2 M NaCl spike was analyzed using Cytiva UNICORN^™^ 7.6 software to calculate the void volume, height equivalent theoretical plate (HETP), peak asymmetry (A_S_) and number of theoretical of plates (N) of the packed resin bed with and without OMEGA. Column performance criteria were set for peak asymmetry of 0.8–1.6, and HETP <0.1. Compression factor values for packed resin beds were determined using Equation 3 (Gebauer and Tschöp, 2018):

$$\text{CF}=\frac{\text{GSV}}{\text{CV}}$$ (3)

Where GSV is the gravity settled volume of the unpacked resin bed, and CV is the column volume of the packed resin bed.

### Determination of resin bed permeability

Permeability is a measure of a resin bed’s ability to allow passage of mobile phase. Permeability (K, darcys) of a resin bed as determined by Darcy’s law (Darcy, 1856) uses the parameters of volumetric flow rate (Q, mL/s), cross-sectional area of the column (CSA, cm^2^), pressure differential (∆CP, N/cm^2^) across the resin bed, resin bed height (L, cm), and the mobile phase viscosity (µ, N*s/cm^2^) according to the following Equation 4:

$$K=\frac{Q*\;\mu \;*\;L}{\text{CSA}\;*\;\Delta \text{CP}}$$ (4)

Resin bed permeability was determined using an ÄKTA™ liquid handler system. To correct for background pressure of the system, an empty 2.6 cm ID column was filled with 0.1 M NaCl, and ∆CP was measured as a function of flow rates from 150–1,000 cm/h. This ∆CP was subtracted from the ∆CP measured when using a column containing a packed resin bed of 30.0 ± 1.0 cm in resin bed height yielding the background corrected ∆CP values reported in tables and figures. Permeability was then calculated according to the equation above and plotted as a function of linear velocity (cm/hr).

### Determination of dynamic binding capacity

Determination of dynamic binding capacity (DBC) was defined as the loaded volume that results in 10% of mAb breakthrough. The theoretical 10% breakthrough calculation methods are detailed in the supplemental methods. With an ÄKTA™ liquid handler system and continuous monitoring of the effluent absorbance at 280 nm (A280), the model mAb was loaded onto packed columns (N = 3) until A280 measurements exceeded the theoretical 10% breakthrough absorbance value (Supplementary Figure S2). The determination of the load mAb mass is detailed in the supplemental methods. To calculate DBC in the context of this study, Equation 5 was used:

$$\text{DBC}=\frac{\text{Mass}\;\text{of}\;\text{Load}\;\text{mAb}\;\left(\text{mg}\right)}{\text{Volume}\;\text{of}\;\text{Resin}\;\left(\text{mL}\right)}$$ (5)

Critically, the gravity settled resin volume was used to determine DBC in place of the packed column volume. This was chosen to account for differences in resin displacement and compression due to OMEGA. The DBC determination using packed resin bed volume in place of gravity settled volume is reported in Supplementary Figure S3. The resultant DBC values between the OMEGA and standard column were tested for statistical significance using an unpaired t-test.

### Antibody purification cycles

Using an ÄKTA™ liquid handler system, cell culture harvest solution containing a model mAb was loaded onto columns packed using Protein A resin with and without OMEGA. The resin load factor used was based on 80% of the experimentally determined DBC. Antibody was eluted off the column with a low pH buffer, collected, and passed through a 0.2 µm filter. Throughout each purification, A280, pH, conductivity, and ∆CP were monitored to assess operational performance.

### Antibody product quality testing

Column eluate was analyzed by high performance liquid chromatography-based size exclusion chromatography (SEC-HPLC) and enzyme linked immunosorbent assay (ELISA) for residual host cell protein quantitation (resHCP). The SEC-HPLC analysis was performed using an Agilent 1,200 series HPLC, a 7.8 × 300 mm TOSOH TSKgel column with a 5 µm mean particle size and 25 nm mean pore size, a mobile phase composed of sodium chloride and sodium phosphate, and UV detection at 280 nm. The resHCP-ELISA was performed using the Cytiva Amersham HCPQuant CHO kit according to manufacturer’s guidance.

## Results

Examination of the operational impact of OMEGA on chromatography performance was completed by packing chromatography columns with Protein A resin, both with and without OMEGA. The impact of OMEGA on packed resin support across column geometries was confirmed by measuring compressibility, linear velocity, ∆CP, and permeability. The effect on resin binding performance was determined by quantifying the DBC of the columns with and without OMEGA. Lastly, the feasibility of OMEGA for use with a biotherapeutic was assessed by purifying a model mAb. Pressures were recorded during the critical steps of loading and eluting biologic material, where failures commonly occur. The purified mAb was subsequently measured for residual host-cell protein content and the amount of protein aggregation.

### Column performance

**FIGURE 2:**
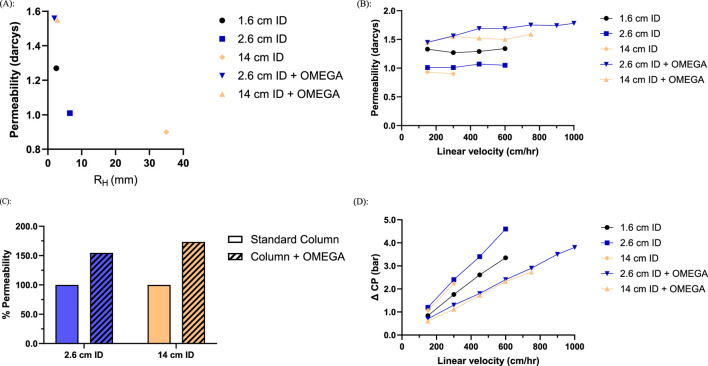
Column geometry and flow characteristics of columns packed with OMEGA and without OMEGA at various inner diameters (n = 1): **(A)** Resin bed permeability at 300 cm/h as a function of hydraulic radius; **(B)** Resin bed permeability at 300 cm/h of columns with OMEGA normalized to standard columns **(C)** Resin bed permeability at assessed linear velocities; **(D)** Pressure differential across the resin bed at assessed linear velocities.

Performance tests for the 2.6 cm ID column showed the presence of OMEGA resulted in a HETP increase of 0.02 cm and 0.08 cm increase for the 14 cm ID scale (Supplementary Table S2). Resin compression factor directly impacts HETP and was measured as 17% lower for the 2.6 cm ID column with the OMEGA insert relative to the no insert column (Supplementary Table S2). When fit with OMEGA both the 2.6 cm ID and 14 cm ID columns have a comparable effective hydraulic radius to the unsupported 1.6 cm ID column (Figure 2a), resulting in an 55% and 73% increase in the observed permeability at 300 cm/h respectively (Figure 2b).

Columns were tested across a range of linear velocities until pressure or instrumentation limits were reached. As linear velocity was increased, both the 2.6 cm ID and 14 cm ID columns without OMEGA encountered instrumentation pressure limits above flow rates of 600 cm/h and 300 cm/h respectively (Figure 2d). The increased permeability from OMEGA enabled both the 2.6 cm ID and 14 cm ID columns to operate at dramatically higher linear velocities than their standard column counterparts (Figure 2c). At a flow rate of 300 cm/h, OMEGA equipped columns demonstrated pressure differences of approximately half the magnitude of the standard columns (Figure 2d). Collectively, the reduction in hydraulic radius for columns utilizing OMEGA demonstrated increased permeability which in turn facilitated greater linear velocities than their standard column counterparts.

### Dynamic binding capacity

**FIGURE 3:**
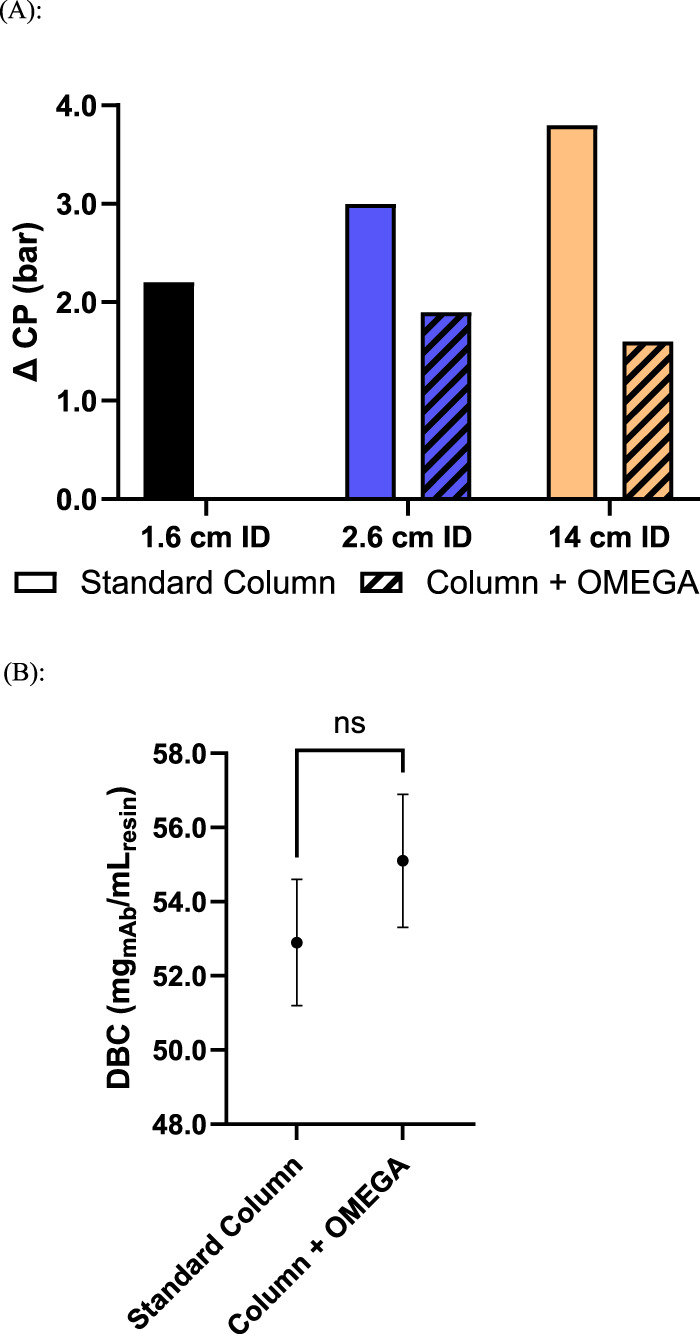
Process performance characteristics of columns packed with OMEGA and without OMEGA at various inner diameters: **(A)** Maximum observed pressure differential across resin bed during elution step of antibody purification (n = 1); **(B)** Dynamic binding capacity, mg of load mAb/mL resin, of Protein A resin, using a basis of gravity settled resin volume (p = 0.2112, N = 3).

To assess the impact of OMEGA on DBC, antibody purification was performed using a 2.6 cm ID column with and without OMEGA, using a solution containing a model mAb. To normalize for displacement of resin volume by the presence of the OMEGA insert, and differences in resin compression factor with and without OMEGA, the gravity settled volume of resin was used for DBC calculations as it represents the available volume of resin to be bound by mAb in each packed column. The average DBC of the column with OMEGA and column without OMEGA did not show statistically significant differences with 55.1 mg of mAb per milliliter of resin and 52.9 mg mAb per milliliter of resin, respectively (Figure 3b). Alternatively, results showing DBC calculated using the packed resin bed volume are shown in the Supplementary Material (Supplementary Figure S3).

### Antibody purification

The performance impact from OMEGA on antibody downstream processing was assessed by performing Protein A chromatography on a clarified cell culture harvest containing a model mAb swith affinity for Protein A. The measured ∆CP during the loading of mAb was 1.1 bar lower at the 2.6 cm ID scale and 1.8 bar lower at the 14 cm ID scale on columns utilizing OMEGA compared to the analogous column without OMEGA at flow rates of 300 cm/h and 255 cm/h, respectively (Supplementary Figure S1). During antibody elution, column flow rates were maintained, and ΔCP measured to be 1.1 and 2.2 bar lower on columns supported with OMEGA versus the standard column counterparts at the 2.6 cm ID scale and 14 cm ID scale, respectively (Figure 3A). No impact was observed to the A280 profile during mAb load runs (Supplementary Figure S4). For the 2.6 cm ID column without OMEGA, pressure limits were exceeded during the 400 cm/h flow rate elution test and unable to reach completion. Across all columns evaluated, similar asymmetry values were measured and HETP values were below the target upper limit of 0.1 cm (Supplementary Table S2). The differences as indicated by SEC-HPLC and residual host-cell protein analysis between the OMEGA and standard column eluates were within the range of typical assay variability. Column yields ranged from 80.0% to 82.9% and eluate column volumes ranged from 1.2 to 1.7 across all column scales with and without OMEGA across the tested flow rates. (Table 1).

**TABLE 1:** Column packing characteristics of a column packed with an inserted OMEGA device and a column with no insert.

Column attributes			Eluate characteristics				
Column	Linear velocity (cm/hr)	Yield (%)	Eluate CV	resHCP (ppm)	SEC-HPLC %HMW	SEC-HPLC %Main	SEC-HPLC %LMW
1.6 ID x 30 BH (cm)	300	82.4	1.2	4420	3.5	96.4	< LOQ of 0.1%
2.6 ID x 30 BH (cm)	200	82.7	1.5	4772	3.3	96.4	0.3
	300	81.9	1.7	4686	3.6	96.2	0.2
2.6 ID x 30 BH (cm) +OMEGA	200	82.4	1.5	4274	3.3	96.4	0.3
	300	82.9	1.3	4605	3.7	96.2	0.1
	400	82.1	1.2	4239	3.4	96.4	0.1
14 ID x 25 BH (cm)	255	80.3	1.4	4974	3.8	96.1	0.1
14 ID x 25 BH (cm) +OMEGA	255	80.0	1.3	4228	3.4	96.4	0.1

## Discussion

In pursuit of mitigating downstream processing chromatography scale-up issues, identifying and characterizing a solution that maintains the flexibility and performance characteristics of resin-chromatography is critical. An ideal solution would reduce ∆CP, maintain DBC, and maintain purification characteristics. As tested, the OMEGA column insert was able to achieve all performance metrics without compromising on the use of existing resin-chromatography equipment.

### Column performance

OMEGA was designed to yield a hydraulic radius comparable to a 1.6 cm ID column regardless of column inner diameter. By modulating the hydraulic radius of the 2.6 cm ID and 14 cm ID columns to match the 1.6 cm ID column typically used for chromatography development, column performance for the larger diameter columns was brought in line with the development-scale column. The predicted effect of hydraulic radius modulation was a decrease in compression and a corresponding increase of measured permeability. As seen in Figure 2, this modulation strategy was effective at increasing resin bed permeability and reducing compression (Supplementary Table S2). In practice, the increased resin bed permeability brings the pressure-flow characteristics of manufacturing-scale column geometries more in line with development-scale columns. Notably, the permeabilities of the 2.6 cm ID and 14 cm ID OMEGA columns were measured to be greater than the 1.6 cm ID column despite identical hydraulic radii (Figure 2a). This may be attributable to the 1.6 cm ID column resin support occurring strictly at the walls of the column, while OMEGA provides support distributed throughout the resin bed. This increase in permeability can be thought of as a trade-off in resin compressibility. The increase of wall-resin contact area may reduce resin compression which may be the cause of the reduction in resolving power (Guiochon et al., 1997; Parental Drug Association, 2008) as indicated by the increase in HETP (Supplementary Table S2). Despite the reduction of resolving power, all columns tested meet the performance threshold of HETP <0.1 cm (Supplementary Figure S5; Supplementary Table S2) (Kim et al., 2023). The difference in support distribution provided by OMEGA could explain the permeability discrepancy, but future studies may be necessary to further understand the effect and how it may impact resolving power for more complex resin purification strategies. Nevertheless, the results support a strategy of maintaining a consistent hydraulic radius across column geometries via OMEGA to increase resin bed permeability and streamline the scale-up of resin chromatography steps from development to manufacturing-scale.

### Antibody purification

To realize the observed pressure and flow rate performance gains when using OMEGA, it was vital to confirm the purified product quality and resin-chromatography performance during purification is preserved when using OMEGA. Critically, these data show the DBC was unimpacted regardless of the inclusion of OMEGA (Figure 3B), indicating that OMEGA does not change the amount of mAb that can be bound per milliliter of resin used as measured by gravity settled volume. When DBC was calculated using the packed resin bed volume a significant difference in DBC is shown between columns with and without OMEGA (Supplementary Figure S3). This discrepancy can be attributed to the difference in resin compression factor (Supplementary Table S2), where the column with OMEGA shows a lower degree of compression. Given the discrepancy in compression between columns with and without OMEGA, the DBC determined using gravity settled volume was considered as the most representative value for this study. During the crucial loading and elution stages of the model mAb purification, the improved pressure-flow characteristics were maintained. Considering the eluate characteristics and the DBCs, columns using OMEGA did not see any adverse effects to binding or elution of the model mAb. These results indicate the additional support provided by OMEGA was effective in reducing resin compression, enabling higher flow rates, and accomplishing both without negative impacts to the quality of product generated (Table 1). Collectively, these results illustrate the readily accessible performance enhancements with OMEGA as well as its suitability for use within biomanufacturing. To generalize the gains seen with OMEGA for batch mode resin chromatography, future work is needed to assess performance impact on resins with alternative ligands, backbone structures, and particle sizes.

### Biomanufacturing industry impact

Within biomanufacturing, upstream operations are increasingly providing improved cost efficacy and product output through greater titers and shorter production cycles. Consequently, downstream operations have become the process bottleneck, struggling to keep pace with upstream production. Using established column hardware with OMEGA and the hydraulic radius control it affords, improved permeability and pressure flow dynamics can be achieved at manufacturing-scale. When used in conjunction with OMEGA, resin-chromatography processes can now achieve flow rates closer to those afforded by membrane-based and monolithic absorbers. This effect may be more pronounced for resins that present challenges for use at the manufacturing-scale due to their susceptibility to compression, such as smaller particle sizes that offer improved resolution during separation, but poor pressure-flow dynamics at larger scales. OMEGA unlocks increased efficiency of resin-chromatography processes by allowing increased flow rates at greater inner diameters. Increases to both resin bed height and inner diameter are viable when using OMEGA without a reduction in process flow rate. An increase to resin bed volume provides the potential for decreasing processing time per cycle and increasing product throughput. A reduction in per cycle processing time and total cycle count would yield reduced manufacturing costs and ultimately lower costs to patients while simultaneously expanding the capacity of downstream operations to better accommodate the gains realized in upstream process intensification. These gains stand to be compounded when used in the context of decreased resin requirements afforded by continuous multicolumn chromatography. Ultimately, OMEGA enhances biomanufacturing process development and intensification by avoiding the need to overhaul existing infrastructure, enabling accelerated manufacturing, and providing avenues to achieve lower costs.

## Data Availability

The original contributions presented in the study are included in the article/Supplementary Material, further inquiries can be directed to the corresponding author.
