# Supplementary material for: Unlocking efficiency in column chromatography with packed bed supporting inserts
Source: Front Bioeng Biotechnol. 2025 Jun 5;13:1613174. doi: 10.3389/fbioe.2025.1613174 (PMC12177508; doi:10.3389/fbioe.2025.1613174)
Supplement: Supplementary file 1 [file DataSheet1.pdf]

Supplementary Material

1 Supplementary Data

Insert Geometry and the Effects on Hydraulic Radius:

Due to the design of the OMEGA insert, there are two unique cross-section aspects to consider: the grid portion of the insert and the pillar supports portion of the insert. These two cross-sectional aspects must be considered independently and are given by the following formulas in which the contributions of each type's unique cross-sectional area and perimeter to hydraulic radius are considered separately:

$$R_H = \frac{\text{Cross-sectional Area of Column - Cross-sectional Area of Grid}}{\text{Perimeter of Column + Perimeter of Grid}}$$

$$R_H = \frac{\text{Cross-sectional Area of Column - Cross-sectional Area of Pillars}}{\text{Perimeter of Column + Perimeter of Pillars}}$$

The portion of the resin bed height that a given cross-section type contributes must be considered to determine a hydraulic radius value for an entire resin bed containing OMEGA inserts. There are three cross-section types: resin bed with OMEGA grid, resin bed with OMEGA pillar, and resin bed with no insert (generally applicable to the amount of resin bed height above the total insert height). To account for the portion of the resin bed height contributed by each of the three types of cross-sections, the resin bed volume (analogous to cross-sectional area) of each type is calculated using the following formulas:

$$\text{Grid Volume} = (\text{Cross-sectional Area of Column - Cross-sectional Area of Grid}) * \text{Bed Height of Grid Cross-section type}$$

$$\text{Pillars} = (\text{Cross-sectional Area of Column - Cross-sectional Area of Pillars}) * \text{Bed Height of Pillar Cross-section type}$$

$$\text{No Insert Volume} = \text{Cross-sectional Area of Column} * \text{Bed Height of No Insert Cross-section type}$$

Whereas the resin bed surface area (analogous to cross-sectional perimeter) of each type is calculated using the following formulas:

$$\text{Grid Surface Area} = (\text{Perimeter of Column} + \text{Perimeter of Grid}) * \text{Bed Height of Grid Cross-section type}$$

$$\text{Pillars Surface Area} = (\text{Perimeter of Column} + \text{Perimeter of Pillars}) * \text{Bed Height of Pillar Cross-section type}$$

$$\text{No Insert Surface Area} = \text{Perimeter of Column} * \text{Bed Height of No Insert Cross-section type}$$

Finally, the sum of volume of each cross-section type can be divided by the sum of surface area of each cross-section type to yield a hydraulic radius of the entire resin bed containing OMEGA:

$$R_H = \frac{\text{Grid Volume} + \text{Pillars Volume} + \text{No Insert Volume}}{\text{Grid Surface Area} + \text{Pillars Surface Area} + \text{No Insert Surface Area}}$$

Determination of Dynamic Binding Capacity

Determination of 10% load mAb breakthrough was performed using Beer's law and the known load mAb material concentration. Beer's law provides absorbance of 100% breakthrough, by taking 10% of this value. The theoretical 10% breakthrough is calculated as shown in the following equation:

$$\text{Theoretical Absorbance at 10\% Breakthrough} =$$

$$\text{Load mAb Conc.} \left(\frac{\text{mg}}{\text{mL}}\right) * \text{Path Length (cm)} * \text{mAb Extinction Coefficient } \frac{\text{mL}}{\text{mg*cm}} * 10\%$$

Load mAb mass used for determination of dynamic binding capacity (DBC) was calculated using the load mAb concentration and load mAb volume. The load mAb volume accounts for the column void volume as determined by the column performance test.

$$\text{Load mAb Mass (mg)} = \text{Load mAb Concentration} \left(\frac{\text{mg}}{\text{mL}}\right) * \text{Load mAb Volume (mL)}$$

Determination of DBC using packed resin bed volume required accounting for the volume of OMEGA inserts to properly account for the true volume of the packed resin bed. This calculation was performed using the following equation.

$$\text{Packed Resin Bed Volume (mL)} =$$

$$\left(\pi * \left(\frac{\text{Inner diameter (cm)}}{2}\right)^2 * \text{Packed bed height (cm)}\right) - \text{Volume of OMEGA inserts (mL)})$$

2 Supplementary Figures and Tables

2.1 Supplementary Figures

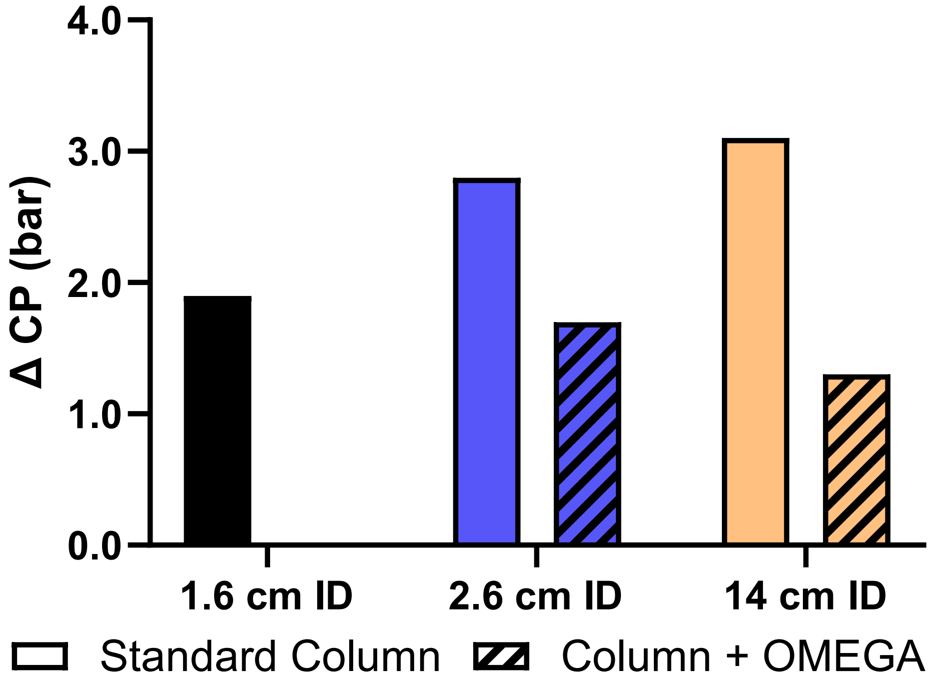

Supplemental Figure 1. Maximum observed pressure differential during loading step of mAb purification.

(A)

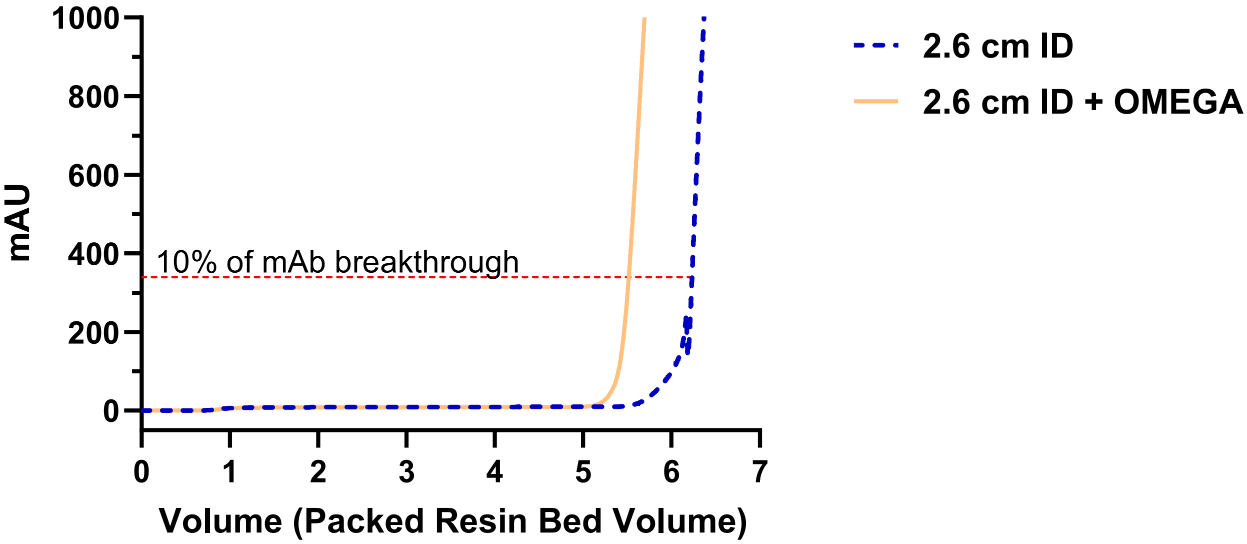

(B)

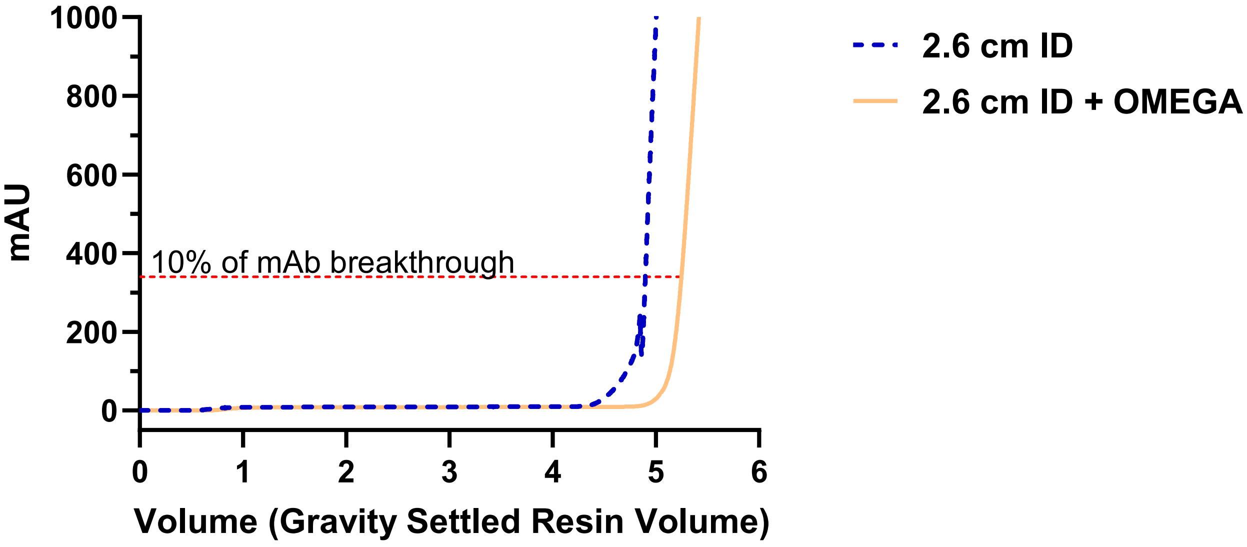

Supplemental Figure 2. Chromatographic A280 traces of dynamic binding capacity loaded to 10% of maximum theoretical breakthrough of load mAb using: (A) A basis of the packed resin bed volume; (B) A basis of the gravity settled resin volume.

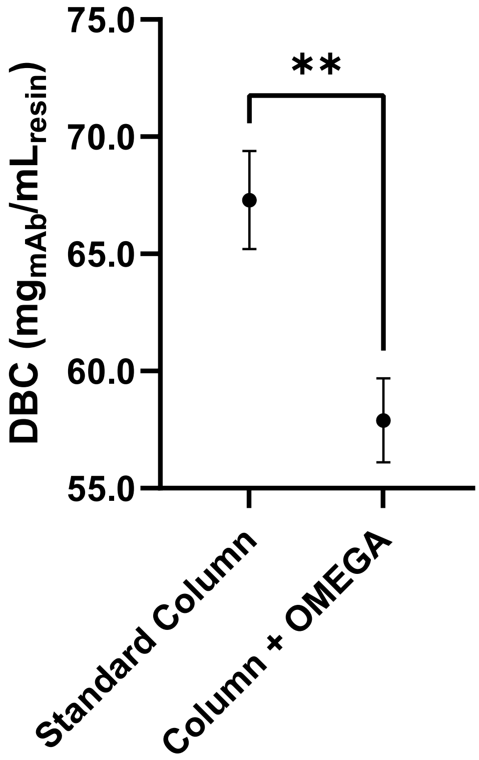

Supplemental Figure 3. Dynamic binding capacity, mg of load mAb/mL resin, of Protein A resin, using a volume basis of packed resin bed volume ($p = 0.0045$, N=3).

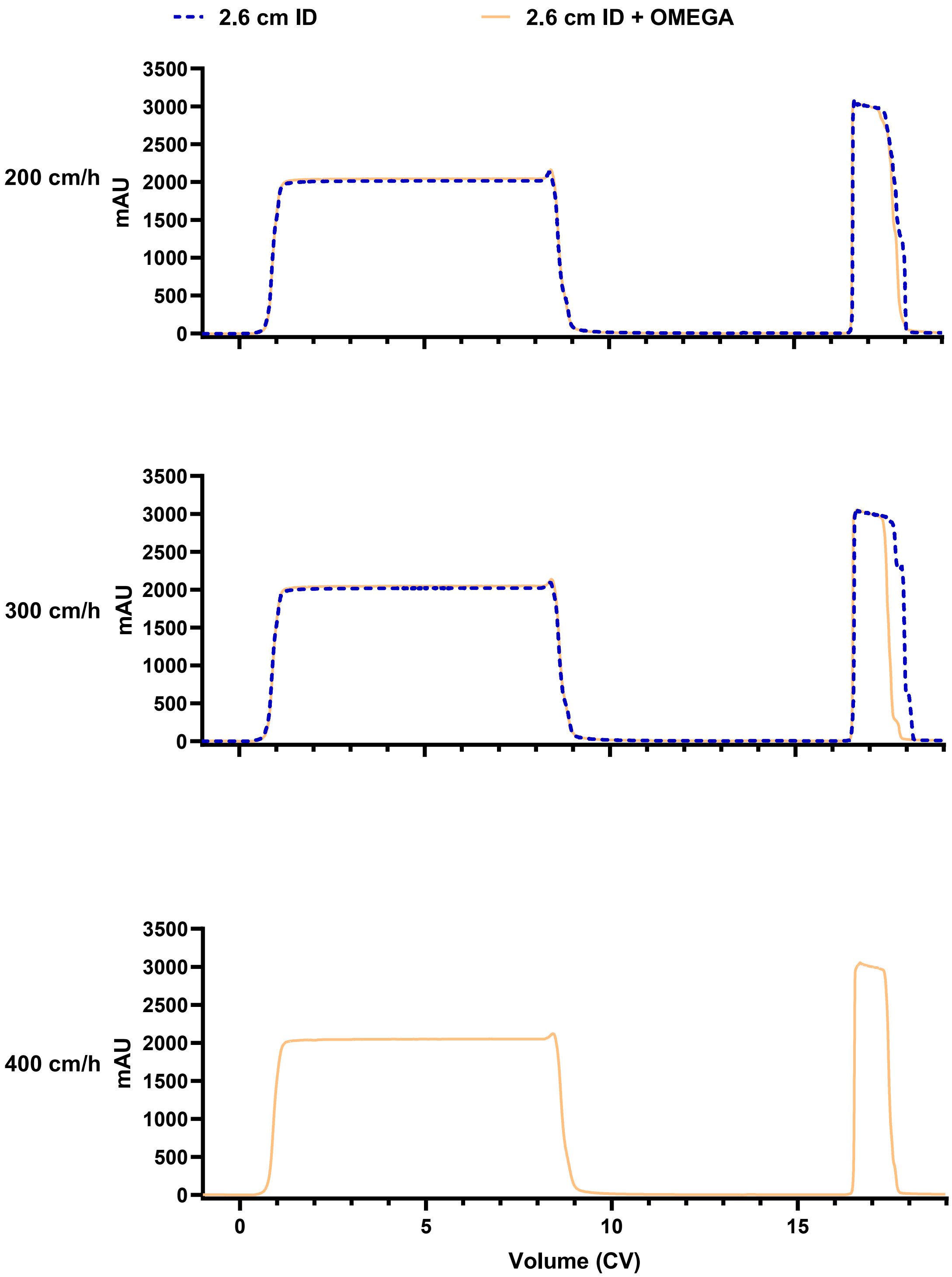

Supplemental Figure 4. Chromatographic A280 traces of antibody purification runs. The 2.6 cm ID column without the OMEGA insert was unable to operate at 400 cm/h due to the pressure limit being exceeded.

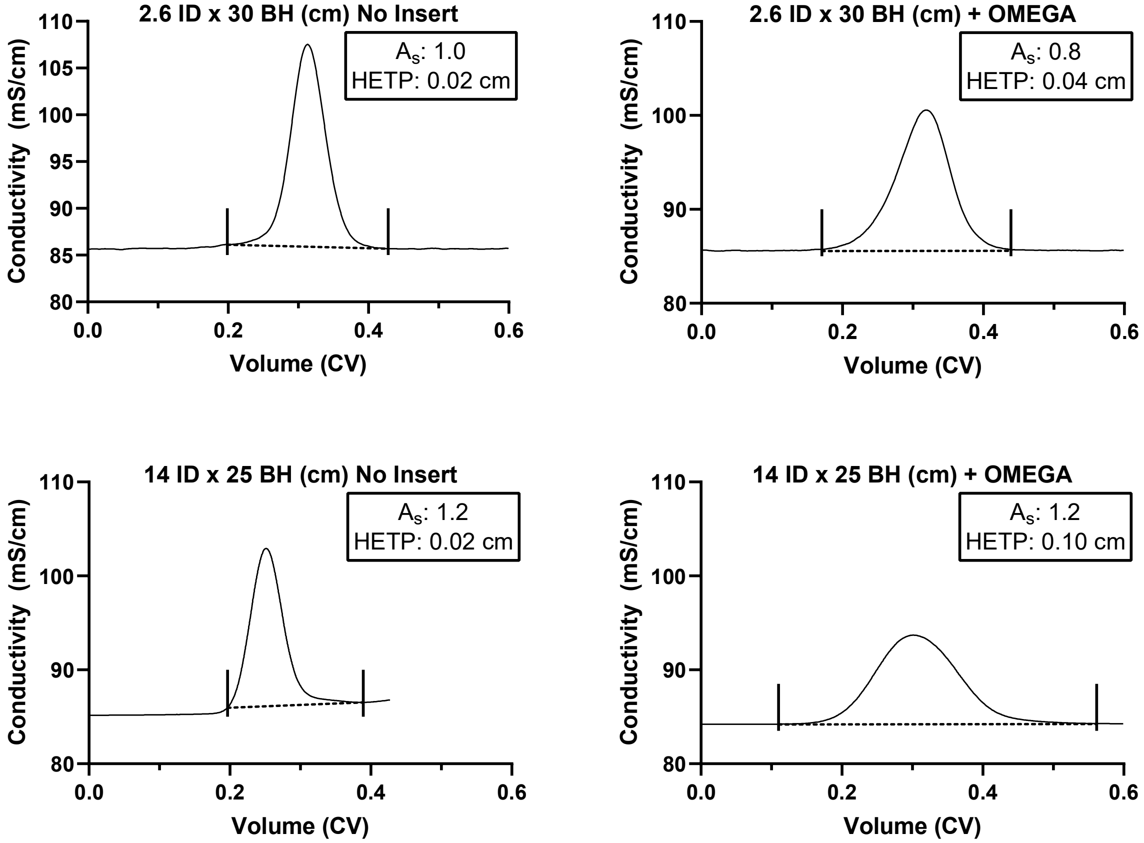

Supplemental Figure 5. Chromatographic conductivity trace of packed resin bed during column performance test. UNICORN™ 7.6 software evaluated the asymmetry and HETP by defining the bounds of the peak within the vertical markers and the area of the peak above the dotted line.

2.2 Supplementary Tables

Column ID (cm)	Standard Column R_H (mm)	Column + OMEGA R_H (mm)
1.6	2.5	N/A
2.6	6.5	1.9
14	35	2.8

Table 1: The effective hydraulic radius and different inner diameters with and without OMEGA.

Column Type	Height Equivalent Theoretical Plates (HETP) (cm)	Peak Asymmetry (A_s)	Compression Factor (CF)
2.6 ID x 30 BH (cm) + OMEGA	0.04	0.8	1.05
2.6 ID x 30 BH (cm) No Insert	0.02	1.0	1.27
14 ID x 25 BH (cm) + OMEGA	0.10	1.2	N/A
14 ID x 25 BH (cm) No Insert	0.02	1.2	N/A

Table 2: Characteristics of packed resin beds with and without OMEGA.
